# Early initiation of breastfeeding among mothers of children under the age of 24 months in Southern Ethiopia

**DOI:** 10.1186/s13006-016-0096-3

**Published:** 2017-01-06

**Authors:** Misrak Getnet Beyene, Nigatu Regassa Geda, Tesfa Dejenie Habtewold, Zuriash Mengistu Assen

**Affiliations:** 1 Ethiopian Public Health Institute, Addis Ababa, Ethiopia; 2 Hawassa University, Hawassa, Ethiopia; 3 University of Saskatchwan, Saskatchwan, Canada; 4 University of Groningen, Groningen, The Netherlands; 5 Addis Ababa University, Addis Ababa, Ethiopia

**Keywords:** Breastfeeding, Timely initiation, Associated factors, Infant/toddler, Cross-sectional study, Ethiopia

## Abstract

**Background:**

The early initiation of breastfeeding (EIBF), or timely initiation of breastfeeding, is the proportion of children put to the breast within one hour of birth. It is an important strategy for reducing perinatal and infant morbidity and mortality, but it remains under practiced in Ethiopia. The aim of the study was to assess the prevalence and the predicting factors associated with EIBF.

**Methods:**

A community based cross-sectional study was conducted in 634 mothers in Dale Woreda, South Ethiopia. Multistage cluster sampling was used to select participating mothers. EIBF was outcome variable whereas sociodemographic characteristics and knowledge and practice of maternal health service were explanatory variables. A face-to-face interview using a pretested semi-structured questionnaire was done from September 2012 to March 2013. To investigate predicting factors, bivariate and multivariate logistic regression analysis was done.

**Results:**

A total of 634 mothers of children under 24 months were interviewed. During the time of data collection, 94.3% of the mothers had breastfed. The prevalence of EIBF was 83.7%. Ownership of the house was a significant predicting factor for EIBF. Mothers who lived in rented houses were significantly less likely (60%) to initiate breastfeeding within one hour of birth compared to mothers who owned their own house: Adjusted odds ratio 0.40 (95% Confidence Interval 0.16, 0.97).

**Conclusion:**

More than three-fourths of mothers initiated breastfeeding within an hour. Findings from our study suggest that improving the mother's socioeconomic status as reflected by house ownership, being a significant predictor of EIBF, would have a central role in improving EIBF.

**Electronic supplementary material:**

The online version of this article (doi:10.1186/s13006-016-0096-3) contains supplementary material, which is available to authorized users.

## Background

The early initiation of breastfeeding (EIBF), or timely initiation of breastfeeding, is the proportion of children born who were put to the breast within one hour of birth [1]. EIBF practice in low and middle-income countries is comparatively higher than in developed countries [2]. A systematic review of studies conducted in Asia, Africa, and South America found the prevalence of EIBF in Ethiopia ranged from 41.6 to 62.6% [3]. Another investigation from fifty-three World Health Organization (WHO) European member states described rates of EIBF as 5 to 84% [4].

Breastfeeding has been universally accepted as the easiest, cost effective and most successful intervention for the satisfactory physical and mental health of children [5–8]. Recent studies in Ethiopia, Ghana, Bolivia and Madagascar found that breastfeeding could prevent 20% to 22% of neonatal deaths [6, 7, 9].

Late initiation of breastfeeding increases the risk of morbidity and mortality such as diarrhea byfivefold [10]. Infectious diseases and malnutrition due to poor breastfeeding practice are major causes of infant death in developing countries [8, 10]. Even though there has been a decrement from 1995 to 2010, the current neonatal mortality rate in Ethiopia is still 29 deaths per 1000 live births [11].

Predicting factors for EIBF include: place of residence and delivery [12]; postnatal care and educational status [9]; unemployment benefit, social welfare and household income [13]; maternal age and socioeconomic status [14]; marital status, smoking, breastfeeding exposure [15]; parity [16]; and antenatal care [17]. Conversely, a prospective study done in India discovered parental education, living condition, number of antenatal visits, birthweight, cultural habit of the population, postnatal breastfeeding advice, previous breastfeeding exposure, and mother’s employment had no significant association with EIBF [18].

Breastfeeding practice is a vital component of primary health care. Realizing the benefit of EIBF, as outlined in the United Nation (UN) sustainable development goals and the WHO millennium development goals, the Ethiopian government developed an infant and young child feeding guideline in 2004. In addition, the government has been implementing the Baby-friendly Hospital Initiative (BFHI) and the community integrated management of childhood illnesses (IMCI) program [19, 20]. However, EIBF is still below the standard recommendation in Ethiopia, perhaps due to the lack of a culturally oriented approach [9]. In addition, predictors of EIBF have not been previously reported in the study area, and this study will go a long way in addressing this gap. The aim of this study was to assess the prevalence and predicting factors of EIBF.

## Methods

### Study setting

The study was conducted in Dale Woreda, Southern Nations Nationalities and Peoples Region (SNNPR). Dale Woreda was selected because of the absence of any previous study, as far as we know, and it is the most densely populated in the SNNP. It is believed that these factors possibly limit the knowledge and practice of EIBF in Dale Woreda. Dale Woreda is located in SNNPR about 326 km south from Addis Ababa, the capital city of Ethiopia. The total area is 302.12 km^2^ with a total population size of 260,767 (132,679 females and 128,088 males). The population density is estimated to be 736 persons per km^2^ and the average land holding is 0.6 ha per person. There are 36 kebeles (smallest administrative unit) with 2087 women headed, and 34,189 men headed households. The Woreda has seven health centers, 29 health posts and one hospital [21].

### Study design and sample

A community based cross-sectional study was conducted in 634 mothers of children under the age of 24 months from September 2012 to March 2013. All mothers who had had a child less than 24 months of age, a term birth, who were permanent residents, and capable of providing informed consent were included. Mothers who were not available during data collection and capable of independent communication were excluded.

### Sample size determination

The required sample size was determined by using a single population proportion formula with the following assumptions:

*n* = required sample size

*p* = prevalence for EIBF, which was 52.4%, in Goba Woreda, Southeast Ethiopia [9].

*d* = marginal error between sample statistics and the population parameter (5%)

*z* = critical value at 95% confidence interval (1.96)

$$ n=\frac{{\left(za/2\right)}^2p\left(1-p\right)}{d^2} $$

$$ n=\frac{(1.96)^2\times 0.524\left(1-0.524\right)}{0.05^2}=\approx 384 $$

Given 1.5 design effect and 10% non-response rate, the final sample size was 634.

### Sampling procedure

**Fig. 1:**
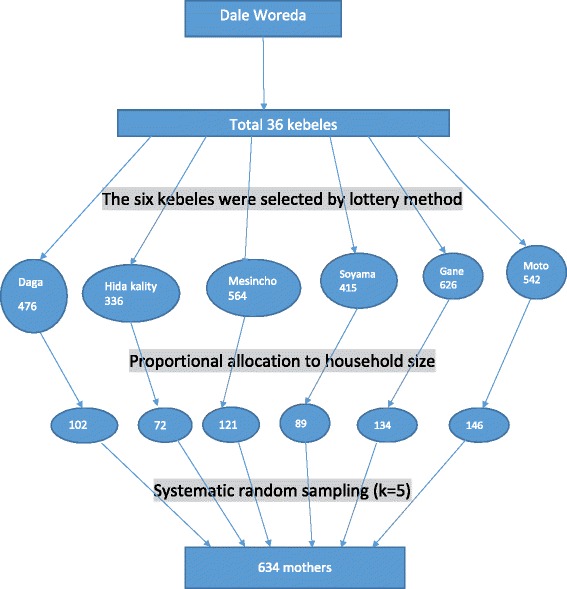
Schematic presentation of the sampling procedure, March 2012

Multistage sampling technique was used for selecting mothers. First, simple random sampling method was used to select six kebeles out of 36 kebeles. Second, total number of mothers (2960) who had children aged less than 24 months was defined by census. Finally, systematic random sampling (sampling interval = 5) was used to select an individual mother (Fig. 1).

### Data collection and instrument

A semistructured questionnaire was developed (MG and GM had a leading role) presuming all the relevant variables were included. First, we identified major indicators of breastfeeding and the related parameters based on previous research evidence, the Ethiopian Demographic Health Survey (EDHS) questionnaire, and WHO infant feeding guidelines. Then, the questionnaire was developed in English and professionals fluent in both languages translated it to Amharic (local language) using backward-forward approach. Next, it was pretested. Finally, redundant, lengthy and vague questions were revised. In addition, probing phrases were added for some of the questions. The questionnaire has three parts; section 1: sociodemographic characteristics of mothers, section 2: knowledge and practice on maternal health service, section 3: breastfeeding practice. An additional word file shows this in more detail (see Additional file 1). During data collection, completeness of filled questionnaire was ensured. A replacement technique from closest household was used whenever an eligible mother was not available for the data collection.

### Variables

EIBF was the outcome variable. Taking into account the definition of WHO [1], initiating breastfeeding within one hour of birth was coded as ‘1’, whereas after one hour of birth was coded as ‘0’ for logistic regression analysis. Sociodemographic characteristics (maternal age, age of child, sex of child, birthweight of child, marital status, educational status, ethnicity, religion, household income) and maternal health service (place of birth, assistance during delivery, health education on breastfeeding, source of information about breastfeeding, antenatal care) were explanatory variables. These explanatory variables were chosen based on previous research evidence, Ethiopia Demographic and Health Survey questionnaire, and WHO infant feeding guidelines.

### Data processing and analysis

Printed frequency was used to checking accuracy, consistency, and missed values of variables. All variables *p*-value ≤ 0.05 in the bivariate logistic regression analysis were included in multiple logistic regression model. Variables *p*-value ≤ 0.05 were considered as an independent predicting factor for EIBF in the final model. The strength of association was described using odds ratio and 95% confidence interval. Statistical Package for the Social Sciences software package (version 16.0) was used to process and analyze all data.

## Results

### Sociodemographic characteristics

A total of 634 mothers of children less than 24 months were interviewed. The mean ± SD age of mothers was 26 ± 5.04 years. The mean ± SD of the age of children was 13 ± 6.31 months (Table 1).

**Table 1:** Sociodemographic characteristics, Dale Woreda, March 2012

Variables	Number (*n*)	Percent (%)
Current age of mother (years)		
15–19 20–24 25–29 30–34 ≥ 35	26 210 223 120 55	4.1 33.1 35.2 18.9 8.7
Age of child in months		
0–5 6–11 12–17 18–23	90 166 187 191	14.2 26.2 29.5 30.1
Sex of child		
Male Female	317 317	50.0 50.0
Birthweight of child		
< 2.5 kg ≥ 2.5 kg Do not know	6 268 360	0.9 42.3 56.8
Marital status		
Married Others^a^	611 23	96.4 3.6
Educational status		
Attended no formal school Attended formal school	145 489	22.9 77.1
Ethnicity		
Sidama Wolayita Amara Oromo Others^b^	364 91 68 37 74	57.4 14.4 10.7 5.8 11.7
Religion		
Christian Muslims	612 22	96.5 3.5
Household monthly income		
0–500 501–1000 1001–1500 1501–2000 > 2000 Didn't know	87 73 19 23 36 396	13.7 11.5 3.0 3.6 5.7 62.5
Ownership of house		
Owner Rented Dependent	374 223 37	59.0 35.2 5.8
Parity		
One 2–4 ≥ 5	240 280 114	37.9 44.2 18.0
Number of living children		
One 2–4 ≥ 5	249 292 93	39.3 46.1 14.7

^a^ Widowed, single, divorced

^b^ Guragge, Tigray, Kembata, Hadiya, Benchimaji

### Knowledge and practice of maternal health service

The main sources of breastfeeding information were health workers (21.5%), husbands/partners (21.9%), grandmothers (28.7%), and friend/neighbours (29.0%). As presented in Table 2, 42.6% of mothers had antenatal care. More than half (56.3%), mothers delivered their babies at home.

**Table 2:** Knowledge and practice of maternal health service, Dale Woreda, March 2012

Variables^a^	Number	Percent (%)
Frequency of listening to radio		
Almost every day At least once a fortnight Less than once a week Not at all	296 60 41 237	46.7 9.5 6.5 37.4
Frequency of reading newspaper		
Almost every day At least once a fortnight Less than once a week Not at all	32 62 99 441	5 9.8 15.6 69.6
Antenatal care		
Yes No	270 364	42.6 57.4
Place of birth		
Outside health facility Health facility	357 277	56.3 43.7
Assistance during delivery		
Health professional Traditional birth attendant No one	286 324 24	45.1 51.1 3.8
Breastfeeding advice/counselling		
Yes No	507 127	80.0 20.0

^a^index child: the infant that the mother currently breastfeeds

### Early initiation of breastfeeding

Five hundred ninety-eight (94.3%) of the mothers had breastfed at the time of data collection. Of those who had breastfed, 517 (83.7%) mothers initiated breastfeeding within one hour after birth.

### EIBF and predicting factors

As presented in Table 3, bivariate test of association, strongly significant predicting factors of EIBF were ethnicity, ownership of the house, number of living children, antenatal care, and place of birth.

**Table 3:** Bivariate association of early initiation of breastfeeding in Dale Woreda, March 2012

Characteristics	Initiation of breastfeeding (*n* = 618)		OR (CI)	*p* - value
	Within 1 h (early) *n* (%)	After 1 h *n* (%)		
Mother's age (years)				
15–19 20–24 25–29 30–34 ≥ 35	17 (70.8) 163 (79.5) 191 (87.2) 98 (85.2) 48 (87.3)	7 (29.2) 42 (20.5) 28 (12.8) 17 (14.8) 7 (12.7)	1 0.6 (0.24, 1.60) 0.4 (0.14, 0.94) 0.4 (0.15, 1.17) 0.4 (0.11, 1.16)	1 0.33 0.03 0.09 0.08
Sex of child				
Male Female	262 (83.7) 255 (83.6)	51 (16.3) 50 (16.4)	1 1.0 (0.65, 1.52)	1 0.97
Birthweight of child				
< 2.5 kg ≥ 2.5 kg Do not know	3 (60.0) 204 (78.2) 310 (88.1)	2 (40.0) 57 (21.8) 42 (11.9)	0.2 (0.03, 1.25) 0.5 (0.31, 0.75) 1	0.08 0.001 1
Marital status				
Married Others^a^	502 (83.9) 96 (16.1)	15 (75.0) 5 (25.0)	1.7 (0.62, 4.91) 1	0.29 1
Educational status of the mother				
Attended no formal school Attended formal school	129 (90.2) 388 (81.7)	14 (9.8) 87 (18.3)	2.1 (1.14) 1	0.02 1
Ethnicity				
Sidama Amara Oromo Wolayita Others^b^	323 (91.0) 49 (73.1) 27 (75.0) 61 (67.0) 57 (82.6)	32 (9.0) 18 (26.9) 9 (25.0) 30 (33.0) 12 (17.4)	1 0.3 (0.14, 0.52) 0.3 (0.13, 0.69) 0.2 (0.11, 0.36) 0.5 (0.23, 0.97)	1 <0.001 0.005 < 0.001 0.04
Religion				
Christian Muslim	500 (83.8) 17 (81.0)	97 (16.2) 4 (19.0)	1 0.8 (0.27, 2.50)	1 0.73
Monthly family income in Ethiopian birr (1US $ =18 ETB)				
0–500 501–1000 1001–1500 1501–2000 > 2000 Didn't know	70 (82.4) 52 (74.3) 16 (94.1) 15 (65.2) 27 (77.1) 337 (86.9)	15 (17.6) 18 (25.7) 1 (5.9) 8 (34.8) 8 (22.9) 51 (13.1)	0.7 (0.38, 1.33) 0.4 (0.24, 0.81) 2.4 (0.31, 18.65) 0.3 (0.12, 0.70) 0.5 (0.22, 1.18) 1	0.28 0.01 0.39 0.01 0.12 1
Ownership of house				
Owner Rented Dependent	334 (91.8) 158 (72.1) 25 (71.4)	30 (8.2) 61 (27.9) 10 (28.6)	1 0.2 (0.14, 0.37) 0.2 (0.10, 0.51)	1 < 0.001 < 0.001
Parity				
One 2–4 ≥ 5	176 (75.5) 236 (86.8) 105 (92.9)	57 (24.5) 36 (13.2) 8 (7.1)	1 2.1 (1.34, 3.36) 4.3 (1.95, 9.26)	1 0.001 < 0.001
Number of living children				
One 2–4 ≥ 5	182 (75.5) 249 (87.7) 86 (92.5)	59 (24.5) 35 (12.3) 7 (7.5)	1 2.3 (1.45, 3.65) 4.0 (1.75, 9.08)	1 < 0.001 0.001
Frequency of listening to radio				
Almost every day At least once a fortnight Less than once a week Not at all	221 (76.7) 44 (80.0) 39 (95.1) 213 (91.0)	67 (23.3) 11 (20.0) 2 (4.9) 21 (9.0)	1 1.2 (0.59, 2.48) 6.0 (1.39, 25.13) 3.1 (1.82, 5.20)	1 0.60 0.02 < 0.001
Frequency of reading newspaper				
Almost every day At least once a fortnight Less than once a week Not at all	22 (71.0) 46 (78.0) 78 (83.0) 371 (85.5)	9 (29.0) 13 (22.0) 16 (17.0) 63 (14.5)	0.4 (0.18, 0.94) 0.6 (0.31, 1.17) 0.8 (0.45, 1.51) 1	0.04 0.14 0.54 1
Antenatal care				
Yes No	206 (78.0) 311 (87.9)	58 (22.0) 43 (12.1)	1 2.0 (1.32, 3.14)	1 0.001
Place of birth				
Outside Health Facility Health facilities	312 (89.7) 205 (75.9)	36 (10.3) 65 (24.1)	2.7 (1.76, 4.30) 1	< 0.001 1
Assistance during delivery				
Health professional Traditional birth attendant No one	211 (75.9) 283 (89.6) 23 (95.8)	67 (24.1) 33 (10.4) 1 (4.2)	1 2.7 (1.73, 4.28) 7.3 (0.97, 55.10)	1 < 0.001 0.05
Breastfeeding advice/counselling				
Yes No	418 (84.8) 99 (79.2)	75 (15.2) 26 (20.8)	1.5 (0.89, 2.41) 1	0.13

^a^Widowed, single, divorced

^b^Guragge, Tigray, Kembata, Hadiya, Benchimaji

After an adjustment for all confounding factors, multivariate test of association in Table 4, living in a rented house was found a significant predicting factor. Mothers who lived in rented houses were significantly less likely (60%) to initiate breastfeeding within one hour of birth as compared to mothers who lived in their own houses (*p*-value = 0.04, OR 0.4, 95% CI 0.16, 0.97).

**Table 4:** Multivariate association of early initiation of breastfeeding and selected variables in Dale Woreda, March 2012

Characteristics	Initiation of breastfeeding (*n* = 618)		OR (CI)	*p*- value
	Within 1 h (early) *n* (%)	After 1 h *n* (%)		
Mother's age (years)				
15–19 20–24 25–29 30–34 ≥ 35	17 (70.8) 163 (79.5) 191 (87.2) 98 (85.2) 48 (87.3)	7 (29.2) 42 (20.5) 28 (12.8) 17 (14.8) 7 (12.7)	1 1.6 (0.58, 4.71) 1.7 (0.57, 5.41) 1.2 (0.34, 4.07) 1.0 (0.20, 4.18)	1 0.33 0.32 0.79 0.91
Birthweight of child				
< 2.5 kg ≥ 2.5 kg Do not know	3 (60.0) 204 (78.2) 310 (88.1)	2 (40.0) 57 (21.8) 42 (11.9)	0.6 (0.07, 4.62) 1.5 (0.78, 2.91) 1	0.61 0.21 1
Educational status of the mother				
Attended no formal school Attended formal school	129 (90.2) 388 (81.7)	14 (9.8) 87 (18.3)	0.9 (0.44, 1.98) 1	0.85 1
Ethnicity				
Sidama Amara Oromo Wolayita Others^a^	323 (91.0) 49 (73.1) 27 (75.0) 61 (67.0) 57 (82.6)	32 (9.0) 18 (26.9) 9 (25.0) 30 (33.0) 12 (17.4)	1 0.7 (0.29, 1.50) 0.8 (0.30, 2.23) 0.5 (0.24, 1.09) 1.2 (0.50, 2.86)	1 0.32 0.70 0.08 0.68
Monthly family income in Ethiopian birr (1US $ =18 ETB)				
0–500 501–1000 1001–1500 1501–2000 > 2000 Didn't know	70 (82.4) 52 (74.3) 16 (94.1) 15 (65.2) 27 (77.1) 337 (86.9)	15 (17.6) 18 (25.7) 1 (5.9) 8 (34.8) 8 (22.9) 51 (13.1)	0.8 (0.42, 1.74) 0.8 (0.43, 1.66) 5.8 (0.69, 48.32) 0.6 (0.22, 1.70) 0.7 (0.27, 1.89) 1	0.67 0.63 0.10 0.34 0.51 1
Ownership of house				
Owner Rented Dependent	334 (91.8) 158 (72.1) 25 (71.4)	30 (8.2) 61 (27.9) 10 (28.6)	1 0.5 (0.26, 0.99) 0.3 (0.12, 0.79)	1 0.04 0.01
Parity				
One 2–4 ≥ 5	176 (75.5) 236 (86.8) 105 (92.9)	57 (24.5) 36 (13.2) 8 (7.1)	1 0.9 (0.15, 5.45) 1.6 (0.09, 25.37)	1 0.92 0.76
Number of living children				
One 2–4 ≥ 5	182 (75.5) 249 (87.7) 86 (92.5)	59 (24.5) 35 (12.3) 7 (7.5)	1 2.0 (0.33, 12.3) 1.7 (0.10, 28.8)	1 0.45 0.73
Frequency of listening to radio				
Almost every day At least once a fortnight Less than once a week Not at all	221 (76.7) 44 (80.0) 39 (95.1) 213 (91.0)	67 (23.3) 11 (20.0) 2 (4.9) 21 (9.0)	1 1.2 (0.53, 2.52) 4.7 (1.02, 21.39) 1.6 (0.85, 3.14)	1 0.70 0.50 0.14
Antenatal care				
Yes No	206 (78.0) 311 (87.9)	58 (22.0) 43 (12.1)	1 1.4 (0.86, 2.38)	1 0.16
Place of birth				
Outside health facility Health facilities	312 (89.7) 205 (75.9)	36 (10.3) 65 (24.1)	0.9 (0.20, 3.86) 1	0.86 1
Assistance during delivery				
Health professional Traditional birth attendant No one	211 (75.9) 283 (89.6) 23 (95.8)	67 (24.1) 33 (10.4) 1 (4.2)	1 1.8 (0.43, 7.75) 3.0 (0.25, 36.7)	1 0.41 0.38

^a^Guragge, Tigray, Kembata, Hadiya, Benchimaji

## Discussion

EIBF reduces infant morbidity and mortality and has economic advantage. Even though the benchmark has not been achieved, the Ethiopian government has initiated different infant and child feeding strategies to optimize EIBF. In this study, we sought prevalence and predicting factors of EIBF. To our knowledge, this study was the first in Dale Woreda where almost half million people are living.

In this study, prevalence of EIBF was 83.7%. According to the WHO infant and young child feeding rating on EIBF, a 0–29% prevalence of EIBF is considered as poor, 30–49% as fair, 50–89% as good and 90–100% as very good [22]. Therefore, our result showed that coverage of EIBF in Dale Woreda was good and stands at 83.7%. This finding was similar with cross-sectional study reports [23, 24], but higher than other studies finding in Ethiopia of 52.4–63% [9, 25]. On the other hand, it was lower than the prevalence rate in rural central Ethiopia 92% [25], India 97.5% [26] and Panama 89.8v% [27].

Ownership of the house was found to be an independent significant predicting factor for EIBF but not maternal age, maternal educational status, antenatal care, place of birth, and parity. This is contrary to the systematic review of studies conducted in Asia, Africa, and South America which identified that the place of delivery, maternal self-confidence and self-efficacy, birth attendant, mode of delivery, parity, cultural practices and beliefs, antenatal care, birth interval, infant birthweight, employment status, occupation, educational status, economic status, postnatal advice on breastfeeding, maternal ill health, breast problem, lack of information, and residence, were significant predicting factors for EIBF [3, 28].

Given the above inconsistencies, prevalence and predicting factors of EIBF are substantially different among regions, nations and continents. This might be due to the difference in study population, sample size, sampling procedure, study period, and setting.

### Policy and practice implication

Despite international collaboration, adaption and implementation of program and policy, EIBF is still below the standard recommendation in Ethiopia [9, 19, 20]. As a result, neonatal mortality is high in Ethiopia. This study also revealed that one out of every five children were not breastfed within one hour of birth, even if all children were expected to breastfed within one hour. House ownership was significantly associated with EIBF implying the importance of economic dependence of mothers. It is believed this also limits access to modern health care. Thus, creating job opportunity, building economic capacity of women sustainably, and strengthening the national infant and young child feeding (IYCF) intervention would have immense advantage to increase EIBF and reducing neonatal mortality rate.

### Strength and limitation

The strength of this study: a community-based study which enables to minimize selection bias; and a large number of mothers included perhaps it increases power and external validity of the study. However, this study has certain limitations. Complex sample analysis, to compensate for unequal probability of recruiting samples from the community, was not done. This study was also subjected to potential recall and social desirability bias. Furthermore, this study shares the limitations of cross-sectional studies.

## Conclusions

More than three-fourths of mothers were practicing EIBF. Findings from our study suggest that improving a mothers socioeconomic status as reflected by house ownership is a significant predictor of EIBF and would have a central role in improving EIBF. Future researchers should conduct a longitudinal qualitative and quantitative study on economically disadvantaged breastfeeding mothers.

## Additional file

Additional file 1: Questionnaire. (DOCX 22 kb)

## Abbreviation

IMCI: Integrated management of childhood illness
IYCF: Infant and young children feeding
MDG: Millennium Development Goal
SNNPR: Southern Nations Nationalities and Peoples Region
UNICEF: United Nations Children’s Fund
WHO: World Health Organization

## References

[1] Victora CG, Bahl R, Barros AJ, França GV, Horton S, Krasevec J, Murch S, Sankar MJ, Walker N, Rollins NC, Group TL (2016). Breastfeeding in the 21st century: epidemiology, mechanisms, and lifelong effect. Lancet.

[2] Patel A, Bucher S, Pusdekar Y, Esamai F, Krebs NF, Goudar SS, Chomba E, Garces A, Pasha O, Saleem S, Kodkany BS, Liechty EA, Kodkany B, Derman RJ, Carlo WA, Hambidge K, Goldenberg RL, Althabe F, Berrueta M, Moore JL, McClure EM, Koso-Thomas M, Hibberd PL (2015). Rates and determinants of early initiation of breastfeeding and exclusive breast feeding at 42 days postnatal in six low and middle-income countries: a prospective cohort study. Reprod Health.

[3] Esteves TM, Daumas RP, Oliveira MI, Andrade CA, Leite IC (2014). Factors associated to breastfeeding in the first hour of life: systematic review. Rev Saude Publica.

[4] Bosi AT, Eriksen KG, Sobko T, Wijnhoven TM, Breda J (2016). Breastfeeding practices and policies in WHO European region member states. Public Health Nutr.

[5] Mullany LC, Katz J, Li YM, Khatry SK, LeClerq SC, Darmstadt GL, Tielsch JM (2008). Breast-feeding patterns, time to initiation, and mortality risk among newborns in southern Nepal. J Nutr.

[6] Baker EJ, Sanei LC, Franklin N (2006). Early initiation of and exclusive breastfeeding in large-scale community-based programmes in Bolivia and Madagascar. J Health Popul Nutr.

[7] Edmond KM, Zandoh C, Quigley MA, Amenga-Etego S, Owusu-Agyei S, Kirkwood BR (2006). Delayed breastfeeding initiation increases risk of neonatal mortality. Pediatrics.

[8] World Health Organization (2000). Effect of breastfeeding on infant and child mortality due to infectious diseases in less developed countries: a pooled analysis. WHO collaborative study team on the role of breastfeeding on the prevention of infant mortality. Lancet.

[9] Setegn T, Gerbaba M, Belachew T (2011). Determinants of timely initiation of breastfeeding among mothers in Goba Woreda, South East Ethiopia: A cross sectional study. BMC Public Health.

[10] Ogbo FA, Page A, Idoko J, Claudio F, Agho KE (2016). Diarrhoea and suboptimal feeding practices in nigeria: evidence from the national household surveys. Paediatr Perinat Epidemiol.

[11] Mekonnen Y, Tensou B, Telake DS, Degefie T, Bekele A (2013). Neonatal mortality in Ethiopia: trends and determinants. BMC Public Health.

[12] Dearden K, Altaye M, Maza ID, Oliva MD, Stone-Jimenez M, Morrow AL, Burkhalte BR (2002). Determinants of optimal breast-feeding in peri-urban Guatemala City, Guatemala. Rev Panam Salud Publica.

[13] Flacking R, Nyqvist KH, Ewald U (2007). Effects of socioeconomic status on breastfeeding duration in mothers of preterm and term infants. Eur J Public Health.

[14] Morhason-Bello IO, Adedokun BO, Ojengbede OA (2009). Social support during childbirth as a catalyst for early breastfeeding initiation for first-time Nigerian mothers. Int Breastfeed J.

[15] Tarrant RC, Kearney JM (2008). Session 1: public health nutrition Breast-feeding practices in Ireland. Proc Nutr Soc.

[16] Horii N, Guyon AB, Quinn VJ (2011). Determinants of delayed initiation of breastfeeding in rural Ethiopia: programmatic implications. Food Nutr Bull.

[17] Dhandapany G, Bethou A, Arunagirinathan A, Ananthakrishnan S (2008). Antenatal counseling on breastfeeding–is it adequate? A descriptive study from Pondicherry, India. Int Breastfeed J.

[18] Chudasama RK, Amin CD, Parikh YN (2009). Prevalence of exclusive breastfeeding and its determinants in first 6 months of life: a prospective study. Online Journal of Health and Allied Sciences.

[19] Miller NP, Amouzou A, Tafesse M, Hazel E, Legesse H, Degefie T, Victora CG, Black RE, Bryce J (2014). Integrated community case management of childhood illness in Ethiopia: implementation strength and quality of care. Am J Trop Med Hyg.

[20] Labbok MH (2012). Global baby-friendly hospital initiative monitoring data: update and discussion. Breastfeed Med.

[21] Wikipedia. Dale (woreda). 2015; Available at: https://en.wikipedia.org/wiki/Dale_(woreda). Accessed 8 Oct 2016.

[22] Berde AS, Yalcin SS (2016). Determinants of early initiation of breastfeeding in Nigeria: a population-based study using the 2013 demograhic and health survey data. BMC Pregnancy Childbirth.

[23] Hailemariam TW, Adeba E, Sufa A (2015). Predictors of early breastfeeding initiation among mothers of children under 24 months of age in rural part of West Ethiopia. BMC Public Health.

[24] Gultie T, Sebsibie G (2016). Determinants of suboptimal breastfeeding practice in Debre Berhan town, Ethiopia: a cross sectional study. Int Breastfeed J.

[25] Ersino G, Henry CJ, Zello GA (2016). Suboptimal feeding practices and high levels of undernutrition among infants and young children in the rural communities of Halaba and Zeway. Ethiopia Food Nutr Bull.

[26] Jennifer HG, Muthukumar K (2012). A cross-sectional descriptive study was to estimate the prevalence of the early initiation of and exclusive breast feeding in the rural health training centre of a medical college in Tamilnadu, South India. J Clin Diagn Res.

[27] Colombara DV, Hernández B, Gagnier MC, Johanns C, Desai SS, Haakenstad A, McNellan CR, Palmisano EB, Ríos-Zertuche D, Schaefer A, Zúñiga-Brenes P (2015). Breastfeeding practices among poor women in Mesoamerica. J Nutr.

[28] Sharma IK, Byrne A (2016). Early initiation of breastfeeding: a systematic literature review of factors and barriers in South Asia. Int Breastfeed J.

