# Supplementary material for: Early initiation of breastfeeding among mothers of children under the age of 24 months in Southern Ethiopia
Source: Int Breastfeed J. 2017 Jan 6;12:1. doi: 10.1186/s13006-016-0096-3 (PMC5217403; doi:10.1186/s13006-016-0096-3)
Supplement: Additional file 1: — Questionnaire. (DOCX 22 kb) [file 13006_2016_96_MOESM1_ESM.docx]

**Structured questionnaire to assess early initiation of breastfeeding practice among mothers of children age less than 24 months.**

**Consent Form**

My name is __________________________________. We are interviewing mothers of 0-2 years to assess the practice of early initiation of breastfeeding. I am going to ask you some questions that are very important for the programmers in infant/child feeding service to plan improved intervention. Your name will not be written in this form and the information you give is kept confidential. If you do not want to answer, all or some of the questions you do have the right to do so. However, your willingness and support to answer all of the questions would be appreciated and thank you for your response.

**Would you participate in responding to questions in this questionnaire?**

Yes__________________ No____________________

**Name and signature of interviewer who sought consent**____________________

**Section-1: Socio-demographic characteristics of mothers**

| S.No. | **Questions and filters** | **Response coding categories** |
| --- | --- | --- |
| 101 | What is your age?  (in Years) | ___________________ |
| 102 | What is the age your last child? (in months) | ________ |
| 103 | Sex of the last child | 1.female  2.male |
| 104 | What was the birth weight of the last child at birth (Kg) | __________  Do not know----------------99 |
| 105 | What is your marital  status? | Single_________________ 1  Married-------------------------2  Divorced______________ 3  Widowed _____________ 4  Other(specify)__________ 99 |
| 106 | What is your current  educational status | Illiterate_____________________1  Read and write_____________ 2  Primary level (1-6) __________ 3  Secondary level (9-12) _________4  Collage level and above________ 5 |
| 107 | What is your religion | Orthodox____________ 1  Muslim______________ 2  Protestant_____________3  Catholic_____________ 4  Other_______________ 5 |
| 108 | What is your Ethnicity | Amhara_________________1  Oromo________________ 2  Guragi_________________3  Tigray_________________4  Other (specify)_________ 99 |
| 109 | What is the average  monthly income of the  house hold? (in birr) | _______________________  Don’t know.….……99 |
| 110 | Who is the owner of your house? | Owned____________________ 1  Rented____________________ 2  Dependent_________________ 3  Other____________________ 99 |
| 111 | How many children are born alive? | No. Children born alive…… |
| 112 | How many children do you have now? | Number of children…….. |

**Section-2: Knowledge and practice on maternal health service.**

| **S.No** | **Questions & filters** | **Code categories** | **Skip** |
| --- | --- | --- | --- |
| 201 | How frequently read newspaper? | Almost every day----------------- 1  At least once a fortnight-------- 2  Less than once a week -------- 3  Not at all ------------------------- 4 |  |
| 202 | How frequently listen to a radio? | Almost every day------------------1  At least once a fortnight----------2  Less than once a week-----------3  Not at all-----------------------------4 |  |
| 203 | Have you attended antenatal clinic in any health facility at least once while you were pregnant with the last child? | Yes……………………….1  No……..………………...2  Don’t know……………..7 |  |
| 204 | Where did you give birth to the last child? | Own home……….……………1  Gov’tal health facility….……..2  Non gov’tal health facility……3  Private clinic----------------------4  Other (specify)……...…..…….99 |  |
| 205 | Who assisted with the delivery of the last child?  Anyone else?  ∗ probe for the type of person & record all person assisting. | Health professional……..…….1  Trained traditional birth  attendant………………… 2  Untrained traditional birth  attendant…………………… 3  Relatives/friends/neighbours--.4  Other (specify)….…....………..…..….99 |  |
| 206 | Have you ever been informed/advised about breastfeeding while you were pregnant or in the period after delivery of the last child? | Yes……………….…………1  No…………………………..2  Don’t know…………………7 |  |

**Section- 3: Breastfeeding practice**

| **S. No.** | **Questions & filters** | **Code categories** | **Skip** |
| --- | --- | --- | --- |
| 301 | Have you ever breast-fed the youngest child? | Yes…………….…….1  No…………………...2 |  |
| 302 | How soon (first 1 hour) after birth did you first put the child to the breast? | Yes…………….…….1  No…………………...0 |  |
| 303 | If the answer for the above question is ‘yes’ What do you feed to your child within one hour? | 1. water 2. cow milk 3. breast milk 4. butter 5. ‘tena adam’ 6. Others |  |
| 304 | If the answer for the above question is ‘No’, what is the reason for not feeding breast within 1 hour? | 1.Illness of mother  2.knowledge deficit  3.illness of child  4.do not want to feed  5. others------------- |  |
| 305 | Within the first three days after delivery, before your milk began flowing regularly, did you feed the child the fluid that came from your breasts? | Yes……………………..1  No………..……………2  Don’t know…………...99` |  |
| 306 | Are you still breast-feeding the child? | Yes……………...…….1  No……………………2 |  |
| 307 | Do you feed by bottle? | Yes_______________1  No_______________ 2 |  |
| 308 | Whoever gave you help or advice how to start and continue breast feeding your  child? | Health worker _______1  Husband _______2  Own/husband's _______3  Mother_______4  Grandmother _______5 Friends/Neighbors _______6  Mass media _______7  None _______8  Other(specify) _______9 |  |
